# *Ficus carica* L. Attenuates Denervated Skeletal Muscle Atrophy via PPARα/NF-κB Pathway

**DOI:** 10.3389/fphys.2020.580223

**Published:** 2020-12-03

**Authors:** Junxi Dai, Yaoxian Xiang, Da Fu, Lei Xu, Junjian Jiang, Jianguang Xu

**Affiliations:** ^1^Department of Hand Surgery, Huashan Hospital, Fudan University, Shanghai, China; ^2^Key Laboratory of Hand Reconstruction, Ministry of Health, Shanghai, China; ^3^Shanghai Key Laboratory of Peripheral Nerve and Microsurgery, Shanghai, China; ^4^Central Laboratory, Shanghai Tenth People’s Hospital, Shanghai, China; ^5^School of Rehabilitation Science, Shanghai University of Traditional Chinese Medicine, Shanghai, China

**Keywords:** denervated muscle atrophy, *Ficus carica*, NF-κß, PPAR, muscle atrophy, peripheral nerve injury

## Abstract

Treatment options for denervated skeletal muscle atrophy are limited, in part because the underlying molecular mechanisms are not well understood. Unlike previous transcriptomics studies conducted in rodent models of peripheral nerve injury, in the present study, we performed high-throughput sequencing with denervated atrophic biceps muscle and normal (non-denervated) sternocleidomastoid muscle samples obtained from four brachial plexus injury (BPI) patients. We also investigated whether *Ficus carica* L. (FCL.) extract can suppress denervated muscle atrophy in a mouse model, along with the mechanism of action. We identified 1471 genes that were differentially expressed between clinical specimens of atrophic and normal muscle, including 771 that were downregulated and 700 that were upregulated. Gene Ontology (GO) and Kyoto Encyclopedia of Genes and Genomes (KEGG) pathway analyses revealed that the differentially expressed genes were mainly enriched in the GO terms “structural constituent of muscle,” “Z disc,” “M band,” and “striated muscle contraction,” as well as “Cell adhesion molecules,” “Glycolysis/Gluconeogenesis,” “Peroxisome proliferator-activated receptor alpha (PPARα) signaling pathway,” and “P53 signaling pathway.” In experiments using mice, the reduction in wet weight and myofiber diameter in denervated muscle was improved by FCL. extract compared to saline administration, which was accompanied by downregulation of the proinflammatory cytokines interleukin (IL)-1β and IL-6. Moreover, although both denervated groups showed increased nuclear factor (NF)-κB activation and PPARα expression, the degree of NF-κB activation was lower while PPARα and inhibitor of NF-κB IκBα expression was higher in FCL. extract-treated mice. Thus, FCL. extract suppresses denervation-induced inflammation and attenuates muscle atrophy by enhancing PPARα expression and inhibiting NF-κB activation. These findings suggest that FCL. extract has therapeutic potential for preventing denervation-induced muscle atrophy caused by peripheral nerve injury or disease.

## Introduction

Peripheral nerve injury often leads to skeletal muscle atrophy, which seriously affects normal limb function and the quality of life of patients (Burns et al., 2012; Ehmsen and Höke, 2020). At present, there are limited clinically effective treatments for reversing or delaying the process of muscle atrophy; however, clarifying the underlying mechanisms can lead to the development of effective therapeutic strategies.

Denervation causes a series of biochemical and physiologic alterations in muscle, resulting from changes in gene expression (Shen et al., 2019). The gene expression profile associated with denervated muscle atrophy has been examined by microarray analysis or high-throughput RNA sequencing (RNA-seq) (Kunkel et al., 2011; Ebert et al., 2012; Jian et al., 2018), which has identified numerous genes such as muscle RING finger 1 (Furlow et al., 2013), high-mobility group box-1 (Yang et al., 2018), and microRNAs including miR-206 (Huang et al., 2016), miR-21 (Soares et al., 2014), and miR29b (Li et al., 2017) that mediate muscle degeneration. It is worth noting that these previous studies were mostly carried out using rodent models of muscle atrophy induced by sciatic nerve transection. Other than investigations on amyotrophic lateral sclerosis and spinal muscular atrophy, there have been no published microarray or RNA-seq studies to date using denervated atrophic muscle from humans (Birger et al., 2019; Onodera et al., 2020).

Oxidative stress plays an important role in the process of muscle atrophy (Scalabrin et al., 2019; Shen et al., 2019; Odeh et al., 2020). Increased production of reactive oxygen species (ROS) in atrophic muscle can lead to oxidative stress along with mitochondrial dysfunction and cellular damage (Muller et al., 2007; Pollock et al., 2017; Scalabrin et al., 2019), and can activate or inactivate transcription factors, metabolic enzymes, and membrane channels (Winterbourn and Hampton, 2008). Inflammation also contributes to the physiologic adaptation of skeletal muscle to denervation (Ma et al., 2018; Shen et al., 2019; Wu et al., 2019). Atrophic muscles have elevated levels of proinflammatory cytokines such as tumor necrosis factor (TNF)-α, interleukin (IL)-1, and IL-6 (Cea et al., 2013; Ma et al., 2019; Wu et al., 2019). Local infusion of recombinant murine IL-6 was shown to induce muscle atrophy in rats (Wu et al., 2019), and inhibition of IL-6 signaling alleviated the severity of muscle atrophy (Cánoves et al., 2013). Thus, drugs with anti-inflammatory and antioxidant effects can potentially prevent muscle atrophy.

*Ficus carica* L. (FCL.) is a flowering plant that contains flavonoids, psoralen, and bergapten and has antioxidant, anti-inflammatory, and antiapoptotic properties (Ali et al., 2012; Badgujar et al., 2014; Melisa et al., 2014; Zhang et al., 2019). FCL. was shown to enhance the levels of the antioxidant enzymes superoxide dismutase and glutathione peroxidase in the serum and liver of diabetic mice and block apoptosis in pancreatic β-cells (Zhang et al., 2019). However, the effect of FCL. on denervated muscle is unknown.

To address this point, in this study, we carried out high-throughput transcriptome sequencing of human muscle samples obtained from four patients with brachial plexus injury (BPI) in order to identify critical pathways and genes related to the atrophy of denervated muscle, and thus gain insight into the molecular basis of this process. As a secondary aim, we examined whether FCL. extract can reverse or delay muscle atrophy in a mouse model along with the mechanism of action.

## Materials and Methods

### Human Muscle Tissue Sample Collection

All experiments of this study were performed in accordance with the guidelines of the Ethics Committee on Human and Animal Experiments (Huashan Hospital, Fudan University). Human atrophic and normal muscles were obtained from four donors diagnosed with BPI, including two with total BPI, one with upper and middle trunk BPI, and one with upper trunk BPI. Inclusion criteria were as follows: (1) total BPI or upper back injury diagnosed by preoperative physical examination and electromyography; (2) uninjured accessory nerve and second to fourth cervical nerve; (3) 18–50 years old; (4) no other diseases; and (5) willing to participate in this study. For the characteristics of these patients, see Supplementary Table 1. Pieces of denervated biceps muscle and normal (non-denervated) sternocleidomastoid muscle about 2 cm in length and 1 cm in diameter were resected from each patient during surgery, and preserved in tissue stage solution (Miltenyi Biotec, Gladbach, Germany) until use.

### RNA-Seq

Total RNA was extracted from muscle tissue samples using the miRNA Isolation Kit (mirVana; Thermo Fisher Scientific, Waltham, MA, United States; AM1561) according to the manufacturer’s protocol. RNA integrity was evaluated using the Agilent 2100 Bioanalyzer (Agilent Technologies, Santa Clara, CA, United States), and samples with RNA integrity number ≥ 7 were retained for analysis. Libraries were constructed using the TruSeq Stranded mRNA LTSample Prep Kit (Illumina, San Diego, CA, United States) according to the manufacturer’s instructions, and were sequenced on the Illumina HiSeq X Ten platform, generating 125/150-bp paired-end reads. Index-coded sample clustering was performed using the TruSeqPE Cluster Kit v3-cBot-HS (Illumina) on a cBot Cluster Generation System according to the manufacturer’s protocol. The Illumina HiSeq X platform was used to sequence the library preparations; 125/150-bp paired-end reads and 50-bp single-end reads were generated.

### Bioinformatic Analysis

#### Quality Control and Mapping

Raw data (raw reads) were processed using Trimmomatic (Bolger et al., 2014). Reads containing poly-N and those of low quality were removed to obtain clean reads, which were mapped to the reference genome using hisat2 (Kim et al., 2015).

#### Identification of Differentially Expressed Genes (DEGs) and Gene Ontology (GO) and Kyoto Encyclopedia of Genes and Genomes (KEGG) Pathway Enrichment Analyses

The fragments per kilobase of transcript per million mapped reads value (Roberts et al., 2011) of each gene was calculated using cufflinks (Trapnell et al., 2010), and the read counts of each gene were obtained with htseq-count (Anders et al., 2015). Differentially expressed genes (DEGs) were identified using the DESeq package of R software with the estimateSize Factors and nbinomTest functions. A *P*-value < 0.05 and fold change > 2 or < 0.5 were set as the thresholds for significantly different expression. Hierarchical cluster analysis of DEGs was performed to explore gene expression patterns. Gene Ontology (GO) enrichment and Kyoto Encyclopedia of Genes and Genomes (KEGG) pathway enrichment analysis of DEGs were performed using R based on the hypergeometric distribution (Minoru et al., 2008).

### Animal Procedures

Male C57 BL/6 mice aged 6–8 weeks and weighing 22–25 g (*N* = 24) were purchased from the laboratory animal center of Charles River Laboratories (Beijing, China). Mice were housed in standard cages in a room at 23°C and 50% relative humidity on a 12:12-h light/dark cycle. The mice were randomly assigned to three groups: the mice that received a sham operation (Control group), the denervated mice that were administered with saline (Den-saline group), and the denervated mice that were infused with FCL. (Den-FCL. group). Mice in both denervated groups were subjected to unilateral sciatic nerve transection under anesthesia as previously described (Wu et al., 2019). Briefly, after deep anesthetization, a 0.5-cm-long portion of the sciatic nerve in the right hind leg of the mouse was resected; the two nerve ends were buried in muscle, and the incision was closed using 4-0 absorbable sutures. After sciatic nerve transection, mice in the FCL. group were treated daily with FCL. extract dissolved in 9% saline (0.15 g/ml saline) by intragastric administration (10 ml/kg). Mice in the saline group received the same amount of saline daily.

### Wet Weight

After 21 days, mice were anesthetized and the gastrocnemius muscles of both left and right hind legs were removed and after saline wash then weighed. The wet weight ratio was defined as the muscle weight of the nerve injury side divided by the weight of the contralateral side. Then these muscle samples were stored in 4% paraformaldehyde and at −80°C until use.

### Hematoxylin–Eosin (HE) and Masson’s Trichrome Staining

Biceps and sternocleidomastoid tissue samples from patients and gastrocnemius muscle samples from mice were fixed in 4% paraformaldehyde and embedded in paraffin. The samples were cut at a thickness of 5 μm and the sections were stained with hematoxylin–eosin (HE) (Beyotime, Shanghai, China) and Masson’s trichrome (Beyotime) to evaluate histopathologic changes. The mean diameter of myofibers was determined by blinded analysis using ImageJ software (National Institutes of Health, Bethesda, MD, United States) from five randomly captured images per mouse in each experimental condition.

### Immunohistochemistry

Expression of the proinflammation factors IL-1β and IL-6 in gastrocnemius muscle was detected by immunohistochemistry. The sections were deparaffinized with xylene and rehydrated with ethanol, and antigen retrieval was performed in 0.01 M citrate buffer (pH 6.0) in a pressure cooker, followed by natural cooling to room temperature. The sections were incubated in 0.3% hydrogen peroxide at room temperature for 10 min; goat serum was used to block the sections for 15 min at room temperature, which were then incubated overnight at 4°C with a rabbit polyclonal anti-IL-1 antibody (1:200 dilution) and IL-6 antibody (1:200 dilution) (both from Abcam, Cambridge, United Kingdom) followed by horseradish peroxidase-conjugated goat anti-rabbit IgG antibody (ABclonal, Wuhan, China) for 30 min at room temperature. Immunodetection was performed using diaminobenzidine solution according to the manufacturer’s instructions. After washing, the sections were counterstained, dehydrated, and then coverslipped using neutral gum sealant.

### Quantitative Real-Time PCR (qPCR)

The RNeasy kit (Qiagen, Valencia, CA, United States) was used to extract total RNA from gastrocnemius muscle. cDNA was synthesized using a first-strand cDNA synthesis kit with oligo dT primers (Invitrogen, Carlsbad, CA, United States) and used for quantitative real-time PCR (qPCR) (MJ Research, Waltham, MA, United States). The thermal cycling conditions were as follows: 94°C for 5 min; 35 cycles at 94°C for 30 s, 55°C for 45 s, and 72°C for 1 min; and 72°C for 5 min. Relative expression level of the target gene was calculated using the cycle threshold (Ct) value corrected with that of the β-actin gene (*ACTB*) (relative expression = 2[Ct_sample_ – Ct_ACTB_]). The primers used to amplify the mouse peroxisome proliferator-activated receptor alpha (*PPAR*α) gene were as follows: forward, GTGTGACATCCCGACAGAC and reverse, CTCACTTCCAGAAACACGA.

### Western Blot Analysis

Frozen gastrocnemius muscle samples were homogenized in radioimmunoprecipitation assay buffer containing 1 mM phenylmethylsulfonyl fluoride and Protease Inhibitor Cocktail (Roche Applied Science). Lysates were centrifuged for 20 min at 12,000 × *g* (4°C) and the protein level in the supernatant was quantified with a bicinchoninic acid assay kit (Beyotime). Proteins were separated by SDS–PAGE (Beyotime) and transferred to a polyvinylidene difluoride membrane (Beyotime) that was blocked with 5% non-fat dry milk in Tris-buffered saline at room temperature, followed by incubation with primary antibodies: rabbit anti-PPARα (1:1000; Affinity Biosciences, United States), rabbit anti-NF-κß P65 (1:5000; Abcam, United Kingdom) and anti-phospho-NF-κß P65 (1:5000; Abcam, United Kingdom), and rabbit anti-IκBα antibodies (1:1000; Affinity Biosciences, United States). After three washes, the membrane was incubated with appropriate secondary antibody (Abcam) at room temperature for 1 h. Enhanced chemiluminescence detection reagent and X-ray film were used for protein visualization.

### Enzyme-Linked Immunosorbent Assay (ELISA)

Expression levels of IL-1β and IL-6 in the gastrocnemius muscle of mice were determined with ELISA kits (Multisciences Biotech, Hangzhou, China) according to the manufacturer’s instructions. After measuring optical density at 450 nm, the expression level was calculated from standard curves.

### Statistical Analysis

All data are presented as mean ± SD. One-way ANOVA was studied to analysis and compare data from three groups. Statistical analyses were performed using SPSS v17.0 software (SPSS Inc., Chicago, IL, United States). *P*-values < 0.05 were considered statistically significant.

## Results

### Human Muscle Atrophy

**FIGURE 1:**
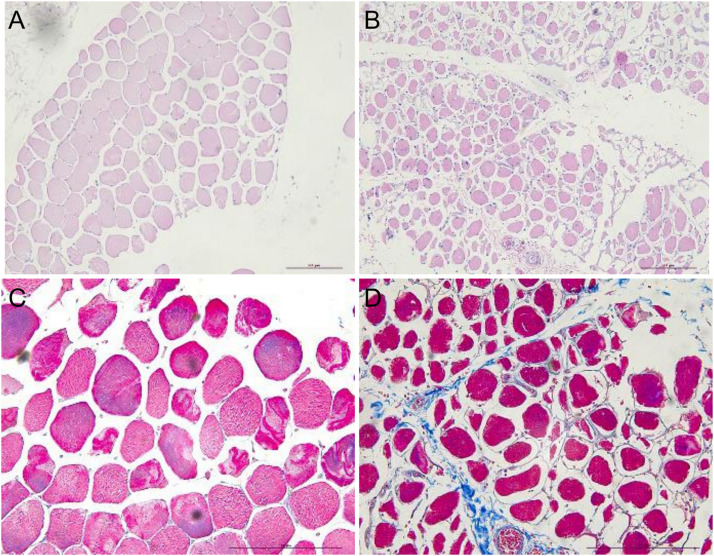
HE and Masson’s trichrome staining showing atrophy of human sternocleidomastoid muscle (left figures) and biceps muscle (right figures). **(A,B)** HE staining showing the irregular arrangement of myocytes in denervated muscle and a smaller stained area compared to normal (non-denervated) sternocleidomastoid muscle. **(C,D)** Masson’s trichrome staining showing the intercellular matrix of denervated muscle covered with blue collagen fiber. Scale bar, 200 μm.

Hematoxylin–eosin staining of sternocleidomastoid muscle tissue sections showed that the myocytes had a regular arrangement with an intact membrane. In contrast, myocytes in denervated biceps muscle had a disordered arrangement and contained numerous vacuoles; Masson’s trichrome staining revealed that the intercellular matrix was covered with collagen fibers, with a much larger stained area than in the sternocleidomastoid muscle (Figure 1).

### DEGs in Denervated Human Muscle

**FIGURE 2:**
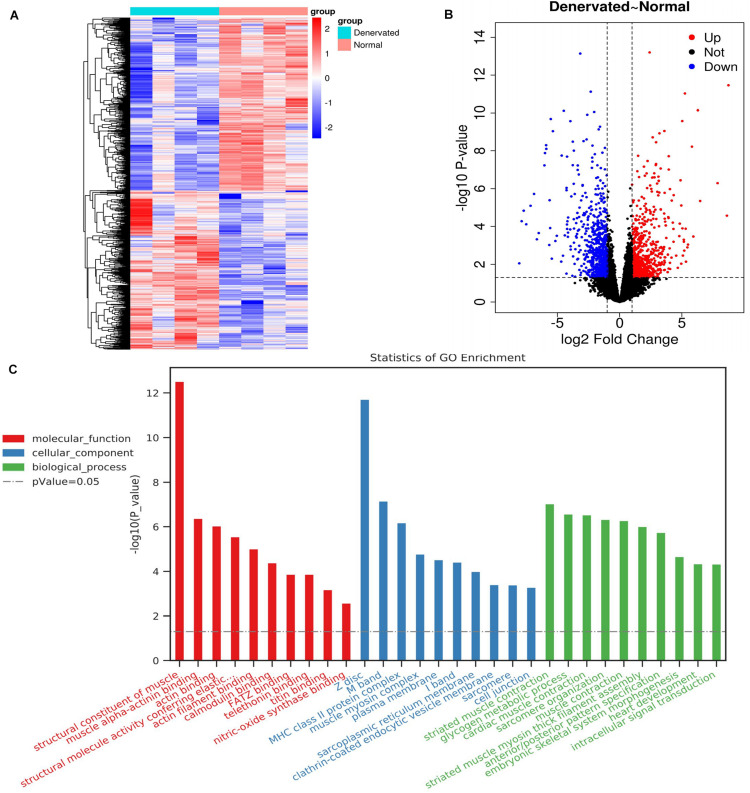
DEGs identified by RNA-seq and associated GO terms. **(A,B)** Heatmap and volcano plot of DEGs. **(C)** Top 30 GO terms.

We identified 1471 DEGs by RNA-seq analysis of atrophic biceps muscle and normal (non-denervated) sternocleidomastoid muscle from patients with BPI, including 771 downregulated and 700 upregulated genes; these are presented as a heatmap (Figure 2A) and in a volcano plot (Figure 2B).

### GO and KEGG Pathways of DEGs

A total of 288 enriched GO terms and 28 KEGG pathways were associated with the identified DEGs. The top 30 enriched GO terms according to the threshold *P*-value (*P* < 0.05) included: (1) molecular function, such as “structural constituent of muscle,” “muscle alpha-actinin binding,” and “actin binding”; (2) cellular component, such as “Z disc,” “M band,” and “MHC class II protein complex”; and (3) biological process, such as “striated muscle contraction,” “glycogen metabolic process,” and “cardiac muscle contraction” (Figure 2C).

**FIGURE 3:**
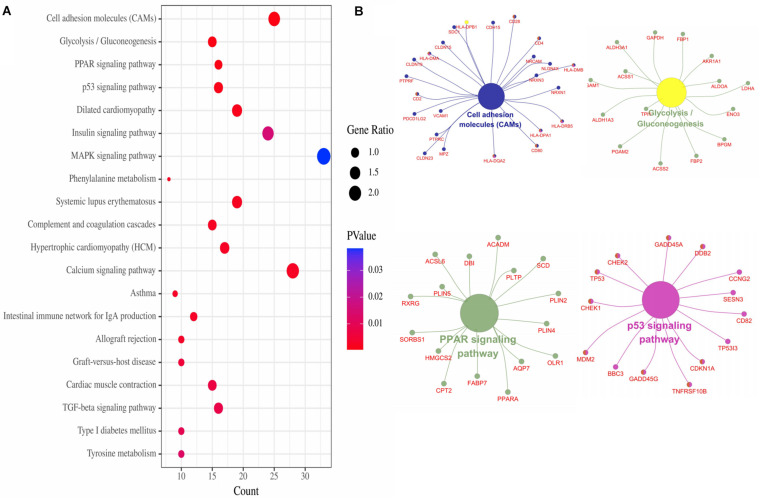
KEGG pathways of DEGs. **(A)** Top 20 KEGG pathways of DEGs. **(B)** Top four pathways gene network.

The top 20 enriched KEGG pathways of the DEGs included “Cell adhesion molecules,” “Glycolysis/Gluconeogenesis,” “PPAR signaling pathway,” “p53 signaling pathway,” “Dilated cardiomyopathy,” “Insulin signaling pathway,” “MAPK signaling pathway,” and so on (Figure 3A). For the gene network of the top four KEGG pathways, see Figure 3B.

### FCL. Attenuates Skeletal Muscle Atrophy

**FIGURE 4:**
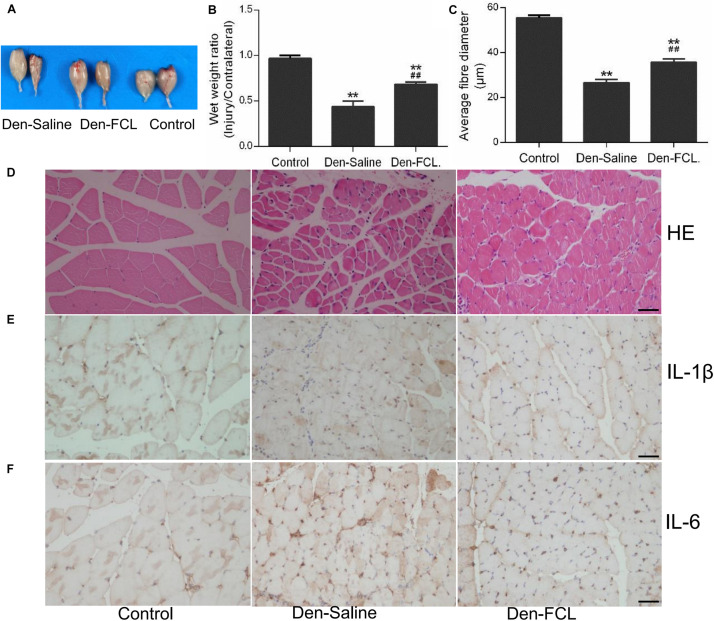
FCL. extract attenuates skeletal muscle atrophy and inhibits IL-1β and IL-6 production in denervated muscle (*n* = 4 mice in each group). **(A,B)** The wet weight ratio of gastrocnemius at 21 days post-denervation. **(C,D)** HE staining of muscle tissue and means ± SEM of fibro-diameter showing muscle atrophy that was reduced by FCL. extract treatment. **(E,F)** Compared to control group muscle, IL-1β and IL-6 positive-expression was increased in the denervated muscle of Den-saline group mice; the levels of both cytokines were lower in the Den-FCL. mice. Scale bar, 50 μm. **p < 0.01 versus Control. ##*p* < 0.01 versus Den-Saline.

After 21 days of FCL. infusion, the denervation-induced loss of muscle wet weight was significantly attenuated, and the mean fibro-diameter of gastrocnemius muscle was larger than in saline-infused mice (Figure 4).

### FCL. Inhibits IL-1β and IL-6 Production in Atrophic Muscles

To determine whether FCL. extract treatment affects the inflammatory response in muscles after denervation, we assessed the expression levels of the proinflammatory cytokines IL-1β and IL-6 in the denervated muscles of mice treated with FCL. extract or saline. Immunohistochemical analysis revealed that IL-1β and IL-6 were upregulated in the denervated muscle of both treatment groups compared to the control group. However, IL-1β and IL-6 positive-expression was lower in the atrophic muscle of mice treated with FCL. extract compared to those treated with saline (Figure 4).

**FIGURE 5:**
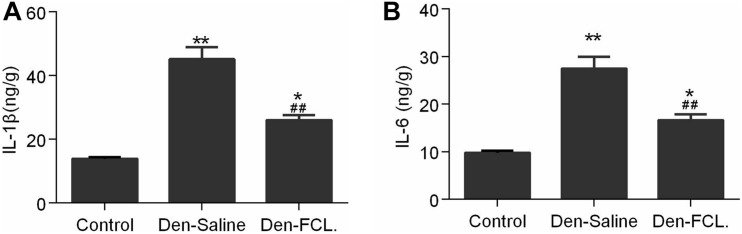
Expression levels of IL-1β and IL-6 in gastrocnemius were detected using ELISA kits. Both IL-1β and IL-6 expressions were increased in the denervated mice muscle, and the elevated level of both cytokines was lower in the Den-FCL. mice (*n* = 4 mice in each group). **(A)** IL-1β expression was determined. **(B)** IL-6 expression was determined. **p < 0.01 and *p < 0.05 versus Control. ##*p* < 0.01 versus Den-Saline.

We also examined IL-6 and IL-1β levels in mouse gastrocnemius muscle by ELISA. Consistent with the immunohistochemistry results, we found that the levels of both cytokines were elevated in atrophic muscle, and were lower in mice treated with FCL. (Figure 5) extract than in those treated with saline. These data suggest that FCL. extract attenuates denervation-induced muscle atrophy by inhibiting the inflammatory response in muscle.

### FCL. Attenuates Skeletal Muscle Atrophy by Stimulating PPARα Expression

**FIGURE 6:**
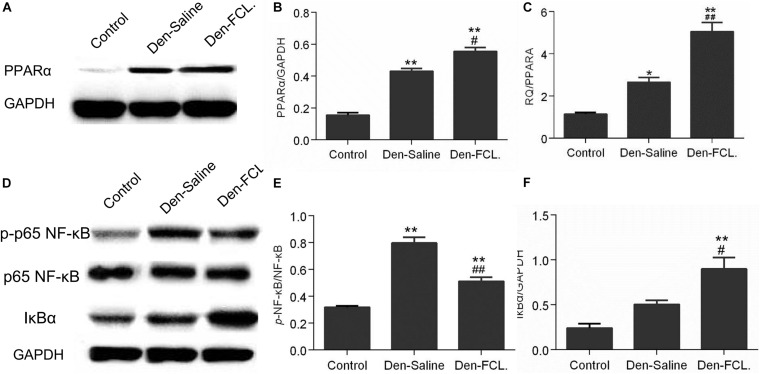
FCL. attenuates skeletal muscle atrophy via PPARα/NF-κB signaling. **(A,B)** Elevated expression of PPARα in both denervated group mice were determined by western blotting. PPARα elevation level of Den-FCL group mice was much higher than the Den-saline group. **(C)** *PPARA* mRNA levels determined by qPCR showed a trend similar to that of the protein. **(D–F)** p65-NF-κB activity was increased after muscle denervation, but was reduced by FCL. extract as compared to saline treatment. Meanwhile, the Den-FCL. group mice showed higher IκBα expression level than other groups (*n* = 4 mice in each group). **p < 0.01 and *p < 0.05 versus Control. ##*p* < 0.01 and #*p* < 0.05 versus Den-Saline.

The RNA-seq results showed that the PPAR signaling pathway is involved in the process of denervated muscle atrophy. To determine whether FCL. extract acts by modulating PPAR signaling, PPARA mRNA and protein expression was evaluated by qPCR and western blotting, respectively (Figure 6). *PPARA* mRNA and PPARα protein levels were upregulated in denervated muscle, with higher levels in mice treated with FCL. extract than in those treated with saline, suggesting that FCL. extract attenuates skeletal muscle atrophy by promoting PPARα expression.

### FCL. Suppresses Inflammation in Atrophic Muscle by Inhibiting Nuclear Factor (NF)-κB Activation

Previous studies have shown that the anti-inflammatory activity of PPARα involves the inactivation of P65-nuclear factor (NF)-κB through the upregulation of inhibitor of NF-κB (IκBα) (Korbecki et al., 2019). To determine whether FCL. extract attenuates denervated muscle atrophy via this mechanism, we evaluated the activation of NF-κB and expression of IκBα (Figure 6). P65-NF-κB activation was increased in denervated muscle; however, this effect was abrogated by FCL. compared to saline infusion. Meanwhile, IκBα expression was higher in Den-FCL. group mice than in Den-saline group. Thus, FCL. attenuates skeletal muscle atrophy by promoting the activation of PPARα and thereby inhibiting NF-κB activity.

## Discussion

A long period of denervation in patients with peripheral nerve injury or disease can lead to skeletal muscle atrophy (Xiong et al., 2012; Ehmsen and Höke, 2020). However, the mechanisms underlying denervated muscle atrophy are not fully understood. Microarray and RNA-seq studies have identified key genes and singling pathways involved in this process (Jian et al., 2018; Shen et al., 2019), but most of the data were from rodent models of peripheral nerve injury and not from human studies.

In the present work, we used high-throughput sequencing to compare gene expression profiles of atrophic biceps muscle and normal sternocleidomastoid muscle from four patients with BPI. Biceps muscle denervation in these patients was confirmed by preoperative physical examination and electromyography. We used the sternocleidomastoid muscle as a control because of the ethical constraint associated with the resection of biceps muscle from the contralateral healthy upper arm of the patient or from a healthy human subject. The sternocleidomastoid muscle was exposed during the surgical process and innervation was confirmed to be intact; we therefore resected this muscle along with the biceps muscle from the same patient in order to reduce heterogeneity across our sample set. We identified 1471 DEGs between denervated and non-denervated muscles, including 700 upregulated and 771 downregulated genes. Most of the enriched GO terms were directly associated with skeletal muscle molecular function and cellular component, such as “structural constituent of muscle,” “Z disc,” “M band,” and “striated muscle contraction”; additionally, other GO terms also included some terms related to the metabolic process, such as “glycogen metabolic process,” “glycogen catabolic process,” and “canonical glycolysis.”

The KEGG pathway analysis showed that the DEGs were mainly enriched in “Cell adhesion molecules,” “Glycolysis/Gluconeogenesis,” “PPAR signaling pathway,” and “p53 signaling pathway.” Cell adhesion is one cell’s autonomous capability of adhering to pluricellular organisms at the basis of the formation of tissues and organs, and cell adhesive processes have be considered as key features of skeletal muscle morphogenesis (Cifuentes et al., 1994; Zhou et al., 2015; Bauer et al., 2019). Kobayashi et al. (1992) have demonstrated that compared with adult mice, the number of neural cell adhesion molecule-positive nerve fibers in motor nerve of sternomastoid was increased in old mice, which show neuromuscular remodeling, indicates that cell adhesion molecules may play a role in regulating the instability of motor nerve terminals. The skeletal muscle accounts for a significant amount of glucose uptake and storage, in pathological conditions, this metabolic regulation ability of skeletal muscle will be perturbed (Deshmukh, 2016; Josep et al., 2016). Thus, lots of pathways related to energy metabolism were enriched in our study, such as “Glycolysis/Gluconeogenesis,” “Insulin signaling pathway,” “Phenylalanine metabolism,” “Type I diabetes mellitus,” and “Tyrosine metabolism.” These findings highlight that the dysfunctional energy metabolism following denervation contribute to muscle atrophy.

The PPAR family comprises three transcription factors—namely, PPARα, PPARβ/δ, and PPARγ—that regulate inflammation and glucose and lipid metabolism by binding to PPAR response elements in the promoter region of target genes (Aleshin and Reiser, 2013; Di et al., 2014; Antonopoulos et al., 2016; Zhong et al., 2017). Recent studies have shown that PPARα encoded by the *PPARA* gene regulates the expression of genes related to inflammation, oxidative stress response, energy metabolism, and mitochondrion and peroxisome function (Sander, 2014; Mitchell et al., 2017; Luo et al., 2020). P53 family members (e.g., tumor protein [TP]53, TP73, and TP63) modulate the expression of Tripartite motif-containing (TRIM)63, which regulates the proteasomal degradation of structural muscle proteins, particularly myofibril components (Ehmsen and Höke, 2020).

Muscle homeostasis depends on the stability of the muscle microenvironment, which is composed of muscle cells (stem cells and interstitial cells), motoneurons, and secreted cytokines (Madaro et al., 2018; Wu et al., 2019). Oxidative stress induces changes in the muscle microenvironment. The release of H_2_O_2_ and ROS from muscle cell mitochondria is increased following denervation (Pollock et al., 2017), which can lead to oxidative stress and consequently, mitochondrial dysfunction, protein degradation, and cellular damage (Qiu et al., 2018; Scalabrin et al., 2019). Inflammation plays an important role in the process of muscle atrophy (Cánoves et al., 2013; Shen et al., 2019; Wu et al., 2019); the enhanced production of proinflammatory cytokines such as IL-1β and IL-6 in atrophic muscle increases ubiquitin expression and the accumulation of ubiquitinated proteins, which promotes protein degradation (Cánoves et al., 2013; Cea et al., 2013; Ma et al., 2019; Ehmsen and Höke, 2020). In our study, IL-1β and IL-6 production was increased in the atrophic muscle of mice at 21 days post-denervation. Given these observations, drugs that can suppress inflammation and oxidative stress may be effective in attenuating and preventing muscle atrophy. FCL. was shown to improve diabetes and inhibit tumor cell proliferation (Umar et al., 2013; Soltana et al., 2019; Zhang et al., 2019) and has known antioxidant, anti-inflammatory, and antiapoptotic effects (Ali et al., 2012; Umar et al., 2013; Melisa et al., 2014; Zhang et al., 2019). We found here that FCL. extract alleviated the severity of denervation-induced muscle atrophy in mice, which was associated with lower levels of IL-1β and IL-6 compared to Den-saline group mice. These results imply that FCL. attenuates muscle atrophy by inhibiting inflammation in denervated muscle.

We observed that p65-NF-κB activity was increased in the denervated muscle of mice. NF-κB is activated during the inflammatory response, which stimulates prostaglandin synthesis (Senf et al., 2008; Korbecki et al., 2019). The regulation of these pathways involves positive feedback but also upregulation of PPARα which prevents the pro-infammatory response from being excessively activated (Delerive et al., 1999; Kono et al., 2009; Korbecki et al., 2019). Meanwhile, according to our RNA-seq findings, PPAR pathway was one of major signaling pathways and the pathway network revealed that PPARA mRNA is only one of three PPAR family genes existing in the DEGs enriched PPAR pathway. To determine whether the attenuation of inflammation in denervated muscle by FCL. extract involves PPARα, we examined *PPARA* transcript and PPARα protein expression and found that both were significantly increased in Den-FCL. group mice compared to Den-saline group mice, suggesting that FCL. extract suppresses the inflammation associated with muscle denervation by promoting of PPARα expression. It is worth noting that PPAR level was increased in denervated muscle regardless of the treatment, possibly reflecting a positive feedback mechanism that counters inflammation induced by denervation (Figure 6).

The anti-inflammatory activity of PPARα involves inactivation of NF-κB, which may involve direct binding of the p65 subunit or enhanced ubiquitination and proteolytic degradation of p65 (Delerive et al., 1999; Korbecki et al., 2019). PPARα has been shown to induce IκBα expression, which blocks the nuclear translocation of NF-κB (Delerive et al., 2000). We observed that IκBα expression was higher while NF-κB activity was lower in FCL. extract-treated mice than in those which were administered saline. Thus, FCL. extract may suppress inflammation by enhancing PPARα expression, which inactivates NF-κB through the upregulation of IκBα.

There are some limitations in our study including: (1) the effects of chemical compositions of FCL remain unclear, which are needed to be confirmed to facilitate reproducibility; (2) the number of human samples is limited; it is necessary for detecting dynamic numbers of DEGs over time-denervation to collect more human denervated samples at various nerve injury timings; and (3) we only determined the effect of FCL. extract at 21 days after denervation in mice, which does not allow for a fine-tuning of the molecular events along the process, and the effect of FCL. extract at other time points is still unknown.

## Conclusion

**FIGURE 7:**
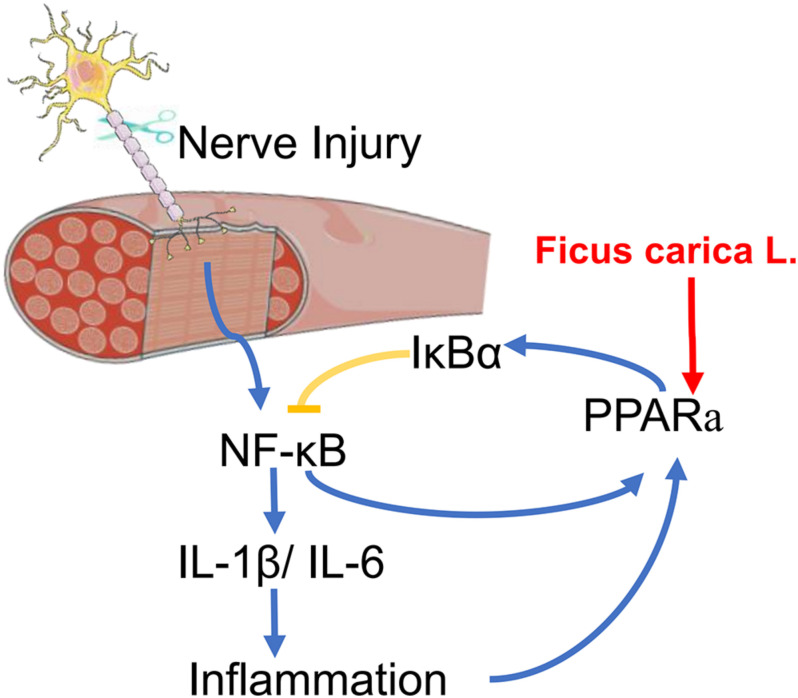
A scheme for FCL. extract attenuating denervated muscle atrophy by inhibiting the inflammation response through PPARα/NF-κB signaling pathways.

In summary, this is the first study to apply transcriptome sequencing to the investigation of denervation-induced muscle atrophy using clinical specimens from patients with BPI. The results presented here provide insight into the molecular basis of denervated muscle atrophy. We also showed that treatment with FCL. extract can delay the atrophy of denervated muscle in mice, which may involve suppression of the inflammatory response via regulation of PPARα and NF-κB signaling (Figure 7). Although additional studies are needed to identify the specific compound(s) in FCL. extract responsible for its protective effects, our findings provide evidence for the therapeutic potential of FCL. extract to delay or prevent denervation-induced muscle atrophy caused by peripheral nerve injury or disease.

## Data Availability Statement

This article contains previously unpublished data. The gene expression dataset is available on NCBI SRA (https://www.ncbi.nlm.nih.gov/sra/PRJNA644778) and the accession number is PRJNA644778.

## Ethics Statement

The studies involving human participants were reviewed and approved by the Ethical Committee, Huashan hospital, Fudan University. The patients/participants provided their written informed consent to participate in this study. The animal study was reviewed and approved by the Institutional Animal Care and Use Committee of Huashan hospital, Fudan University.

## Author Contributions

JJ and JX designed the study. JD, YX, DF, and LX performed the experiments. JD and YX analyzed the data. JD wrote the manuscript. YX made the figures. All authors contributed to the article and approved the submitted version.

## Conflict of Interest

The authors declare that the research was conducted in the absence of any commercial or financial relationships that could be construed as a potential conflict of interest.

## Supplementary Material

Supplementary Table 1 The characteristic data of four donors.
