# Supplementary material for: Ficus carica L. Attenuates Denervated Skeletal Muscle Atrophy via PPARα/NF-κB Pathway
Source: Front Physiol. 2020 Dec 3;11:580223. doi: 10.3389/fphys.2020.580223 (PMC7744683; doi:10.3389/fphys.2020.580223)
Supplement: Supplementary Table 1 — The characteristic data of four donors. [file Table_1.DOCX]

| Sample ID | Age(yr) | Sex | BNI Type | Injury Duration Time (month) |
| --- | --- | --- | --- | --- |
| Case1 | 19 | M | Total BNI | 6 |
| Case2 | 21 | M | Upper trank | 3 |
| Case3 | 38 | M | Upper /Middle trank | 14 |
| Case4 | 54 | F | Total BNI | 3 |

Table1：The characteristics of these four patients.
